# The transcriptome-wide N6-methyladenosine (m^6^A) map profiling reveals the regulatory role of m^6^A in the yak ovary

**DOI:** 10.1186/s12864-022-08585-7

**Published:** 2022-05-11

**Authors:** Shaoke Guo, Xingdong Wang, Mengli Cao, Xiaoyun Wu, Lin Xiong, Pengjia Bao, Min Chu, Chunnian Liang, Ping Yan, Jie Pei, Xian Guo

**Affiliations:** Key Laboratory of Yak Breeding Engineering of Gansu Province, Lanzhou Institute of Husbandry and Pharmaceutical Sciences, Chinese Academy of Agricultural Sciences, Lanzhou, 730050 China

**Keywords:** m^6^A, MeRIP-seq, Estrus, Anestrus, Pregnancy

## Abstract

**Background and aim:**

Yak estrus is a seasonal phenomenon, probably involving epigenetic regulation of synthesis and secretion of sex hormones as well as growth and development of follicles. N6-methyladenosine (m^6^A) is the most common internal modification of the eukaryotic mRNA. However, there are no detailed reports on the m^6^A transcriptome map of yak ovary. Therefore, this study aimed to collected the yak ovarian tissues at three different states of anestrus (YO-A), estrus (YO-F), and pregnancy (YO-P), and obtained the full transcriptome m^6^A map in yak by MeRIP-seq.

**Results:**

The HE staining revealed that the number of growing follicles and mature follicles in the ovary during the estrus period was relatively higher than those in the anestrus period and the pregnancy period. The RT-qPCR showed that the expression of *METTL3, METTL14, FTO, YTHDC1* were significantly different across different periods in the ovaries, which suggests that m^6^A may play a regulatory role in ovarian activity. Next, we identified 20,174, 19,747 and 13,523 m^6^A peaks in the three ovarian samples of YO-A, YO-F and YO-P using the methylated RNA immunoprecipitation sequencing (MeRIP-seq). The m^6^A peaks are highly enriched in the coding sequence (CDS) region and 3′untranslated region (3′UTR) as well as the conserved sequence of “RRACH.” The GO, KEGG and GSEA analysis revealed the involvement of m^6^A in many physiological activities of the yak’s ovary during reproductive cycle. The association analysis found that some genes such as *BNC1, HOMER1, BMP15, BMP6, GPX3,* and *WNT11* were related to ovarian functions.

**Conclusions:**

The comparison of the distribution patterns of methylation peaks in the ovarian tissues across different periods further explored the m^6^A markers related to the regulation of ovarian ovulation and follicular development in the yak ovary. This comprehensive map provides a solid foundation for revealing the potential function of the mRNA m^6^A modification in the yak ovary.

**Supplementary Information:**

The online version contains supplementary material available at 10.1186/s12864-022-08585-7.

## Background

Yak (*Bos grunniens*), an endemic cattle species, is capable of adapting to extremely cold and low-oxygen environments and highly resistant to diseases. It is not only an important source of life and economy for herdsmen in the Qinghai Tibet Plateau but also indispensable livestock in the local animal husbandry economy [1]. Yak generally calve once in two years or twice in three years. Yak ovulations are usually manifested by seasonal physiological changes, and the time of ovulation and conception mainly ranging from August to September [2]. It is reported that epigenetic might regulate the pre-ovulatory follicular growth and development, as well as the follicular selection [3]. In the cold season, yak undergo ovarian dysfunction due to the accumulation of the antral follicles and dysfunction of the granulosa cells. This process may be related to steroid hormone biosynthesis and estrogen signaling pathway, or regulated by epigenetic mechanisms [4]. Xia et.al found that a decreased m^6^A level in the zebrafish *(Danio rerio)* not only disrupted the sex hormone synthesis and the gonadotropin signal transduction gene expression, but also impaired the meiosis process of oocytes [5].

N6-methyladenosine (m^6^A) discovered in 1974, constitute the most prominent internal RNA modification [6, 7]. Remarkably, numerous studies have reported m^6^A executed an important regulatory role in animal reproduction. The spermatocytes lacking METTL3 could not reach the pachytene stage of the meiotic prophase, inhibiting the spermatogonial differentiation and the normal initial meiosis [8], inactivating ALKBH5 and leading to sterility in male mice [9]. It was found in zebrafish that METTL3deletion can impair the meiotic process of oocytes [10]. YTHDF2 may regulate mRNA degradation during meiosis and maturation of oocytes in an m^6^A-dependent manner [11]. YTHDC2 plays an important role in the meiotic process of male and female germ cells, and the deletion may lead to infertility or infertility [12]. However, to the best of our knowledge, there are no reports on m^6^A in the yak ovarian tissues.

Despite the early discovery of m^6^A, studies on this modification were hindered by multiple factors. Until the emergence of new sequencing technology, Dominisini and Meyer [11, 13] combined immunoprecipitation technology with next generation high-throughput sequencing (m^6^A-Seq or MeRIP-Seq). Since then, researchers have successfully constructed the m^6^A map for a variety of animals and plants using the MeRIP-Seq. To study the regulatory mechanism of the yak ovary during estrus and pregnancy, and to promote the further study of yak m^6^A, the ovarian tissues of yak in three different states of anestrus, estrus (also known as follicular phase) and pregnancy, respectively, were collected and the novel full transcriptome m^6^A map in yak was obtained by MeRIP-Seq. By comparing the distribution patterns of the different m^6^A peaks in different ovarian tissues, the relationship between the regulation of the gene expression by m^6^A modification and ovarian function was further explored.

## Results

### Observation of ovarian tissue section

**Fig. 1:**
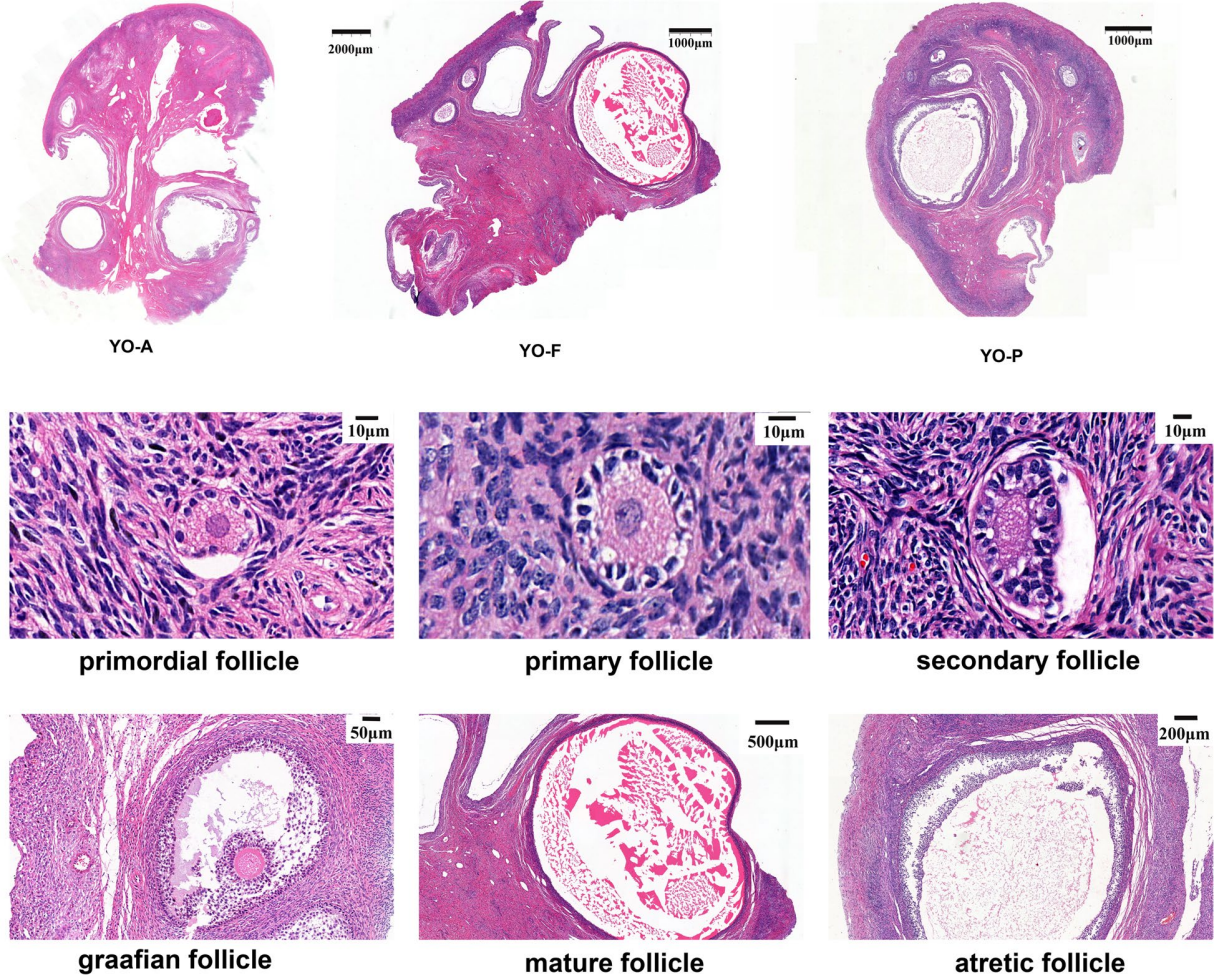
The microscopic observation of the tissue sections of the yak ovaries. YO-A, YO-F, and YO-P separately represent the yak ovary in the anestrus period, follicular (estrus) period, and pregnancy period

The ovarian tissue slices in the anestrus (YO-A), follicular (YO-F), and pregnancy (YO-P) phases were observed and scanned under the microscope using the CaseViewer software. Based on the classification of follicles on the ovarian surface by Yu Sijiu [14], the ovarian capsule was found to be intact, with primordial, primary follicles, secondary, and mature follicles evident in the cortex, and the development of the follicles were normal at all levels (Fig. 1). The fibrous connective tissue in the medullary area was arranged closely without obvious edema or necrosis, and a small number of lymphocytes were infiltrated. By comparison, it was found that there were more growth follicles and mature follicles in August, while in April and December, the number of mature follicles was less. The arrangement of follicular cells in the granulosa layer of primary or secondary follicles was disordered in some tissues. In terms of the volume of follicles, the volume of atresia follicles is larger in August. The diameter of atresia follicles is mostly 2–3 mm, and the largest being up to 3.6 mm. The atretic follicles observed in April were slightly smaller, with a diameter of 0.5–1.5 mm.The diameter of atretic follicles evident in December was between 1–3 mm.

### Comparison of the m^*6*^A enzyme expression level

**Fig. 2:**
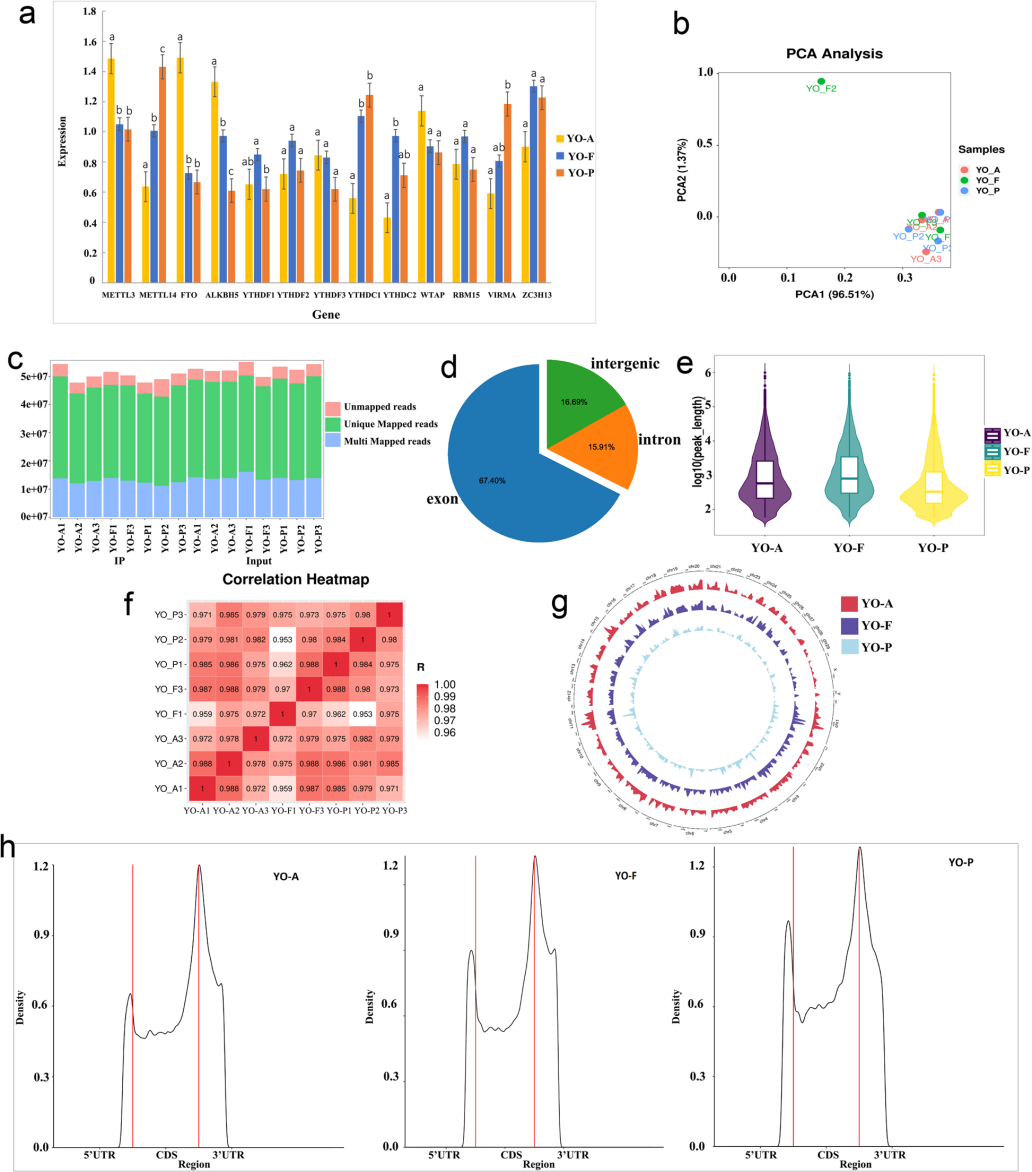
The results of m^6^A methylase expression and genome sequencing. **a** The gene expression levels of 13 common m^6^A methylases on the three groups of YO-A, YO-F and YO-P. Different lowercase letters between two groups represent significant differences (*p* < 0.05), and the same letters among groups represent no significant differences (*p* > 0.05). **b** The principal component analysis of the three groups of samples. **c** The distribution of different types of reads in each sample was counted. **d** The valid data were matched to the reference genome defined as matched to exon, intron and intergenic. **e** The length distribution of m^6^A peaks in the YO-A, YO-F, and YO-P groups. **f** The relative expression levels of IP in the three groups of ovarian samples. **g** The distribution of m^6^A peak sites in the yak genome was obtained from three different samples. The outermost circle represents the chromosome distribution on the genome, the red circle represents the YO-A group, the purple circle represents the YO-F group, and the blue circle represents the YO-P group. **h** Density distribution of the m^6^A peaks in YO-A, YO-F and YO-P transcripts in the different gene structures

In order to further determine whether m^6^A plays a regulatory role in yak ovarian activity, the gene expression of 13 m^6^A-related enzymes: METTL3, METL14, WTAP, FTO, ALKBH5, YTHDF1-3, YTHDC1-2, RBM15, VIRMA, and ZC3H13 were detected using the RT-qPCR. Figure 2a exhibits that the expression levels of METTL3, FTO, and ALKBH5 in the anestrus ovaries are significantly higher than those of the ovaries during estrus and pregnancy (*p* < 0.05). However, the expression level of YTHDF1 in estrus ovaries is significantly higher than that in pregnancy (*p* < 0.05), and the expression levels of YTHDC1 and YTHDC2 in estrus ovaries are significantly higher than those in anestrus (*p* < 0.05). The expression levels of METTL14 and VIRMA in the ovaries during pregnancy is significantly higher than that in anestrus (*p* < 0.05).It can be seen from the test results that there are significant differences in the expression of these methylases at different stages of the ovary. Therefore, we speculate that m^6^A plays an important role in ovarian activity in yaks.

### Sequencing quality control and reference genome alignment

To obtain the m^6^A transcriptome map of the yak ovary, the ovarian tissues in three different periods- YO-A, YO-F, and YO-P were detected by MeRIP-Seq. The samples were obtained from three yaks during each period. The result of the principal component analysis showed that the YO-F2 had poor reproducibility for the same group of samples (Fig. 2b), therefore, the sample data would not be analyzed in the future. The reading with connector, low quality readings (the number of bases with a quality value of Q ≤ 10 accounts for more than 20% of the total read), and low quality bases were filtered out by the Trimomatic v0.36 tool. In the MERIP-seq library, the three groups of YO-A, YO-F, and YO-P yielded an average of 51,426,911, 51,743,067, and 50,341,117 original data readings, and an average of 50,785,722, 51,071,937 and 49,348,875 valid data readings. The proportion of valid readings is above 90% (Supplementary table S1). Unlike m^6^A-seq, which was found in early mammals [11] (11 million to 24 million readings per sample) and rice [15] (23 million to 47 million readings per sample), the technology for m^6^A-seq in the current study was significantly improved and all the sequences have high depths.

The preprocessed valid data was compared to the reference genome (LU_Bosgru_v3.0) by the software Hisat2. The number of reads that can be matched to the genome accounts for about 90% of the data after quality control, among which the number of reads that can only be matched at one location of the genome accounts for 62–67%, while the number of reads that can be matched to multiple locations of the genome accounts for 22–29% (Supplementary table S1), as shown in Fig. 2c. According to the regional information of the reference genome, the valid data that can be matched to the reference genome can be defined as those matched to the exon, intron, and intergenic regions. It can be seen from Fig. 2d that the percentage content of sequencing the sequence localization in the exon region is the highest.

### Identification of the genome-wide m^*6*^A peak

The readings obtained from the yak samples were compared with the yak reference genome. These peaks were annotated using the R package, ChIPseeker after peak scanning the whole gene (Supplementary table S2). Referring to the previous studies, highly credible peaks were determined for each experimental condition (one inbred line under one environmental condition). In short, by crossing the peak regions in pairs between all the three replicates, at least two overlapping regions in the three replicates were designated as the high confidence m^6^A peak regions. About 20,174, 19,747, and 13,523 m^6^A peaks were separately identified in the Yo-A, Yo-F, and Yo-P ovary samples. All the peaks had total lengths of 95,594,574 bp, 122,175,034 bp, and 53,590,770 bp, respectively, accounting for 3.37%, 4.31%, and 1.89% of the genome. The average lengths of m^6^A peaks in the YO-A, YO-F, and YO-P groups were 4738.50 bp, 6,187.02 bp, and 3962.94 bp, the length distribution has been shown in Fig. 2e. The heat map shows the relative expression levels of IP in the three groups of ovarian samples, with similar performance in the three samples of the same group (Fig. 2f). Interestingly, the distribution trend of m^6^A methylation sites in the yak genome of the three different samples was consistent (Fig. 2g). Chr 20 contains more m^6^A sites, followed by chr 8, and chr 23 contains the least m^6^A sites. Due to this, this result is slightly different from the identification result obtained in the rat brain tissue [16]. Each gene or transcript contains 1–3 m^6^A peaks, and more than half of the methylated mRNAs contain only one m^6^A peak. To analyze the topological pattern of m^6^A in the yak ovary, the gene distribution of all identified m^6^A peaks in YO-A, YO-F, and YO-P transcripts was statistically analyzed. It was found that the m^6^A peak was highly enriched in the coding sequence (CDS) region and 3′ untranslated region (3′ UTR) (Fig. 2h). This feature indicates that the distribution of m^6^A methylation in the gene structure of organisms is highly conserved.

### Identification of the differentially methylated RNAs

**Fig. 3:**
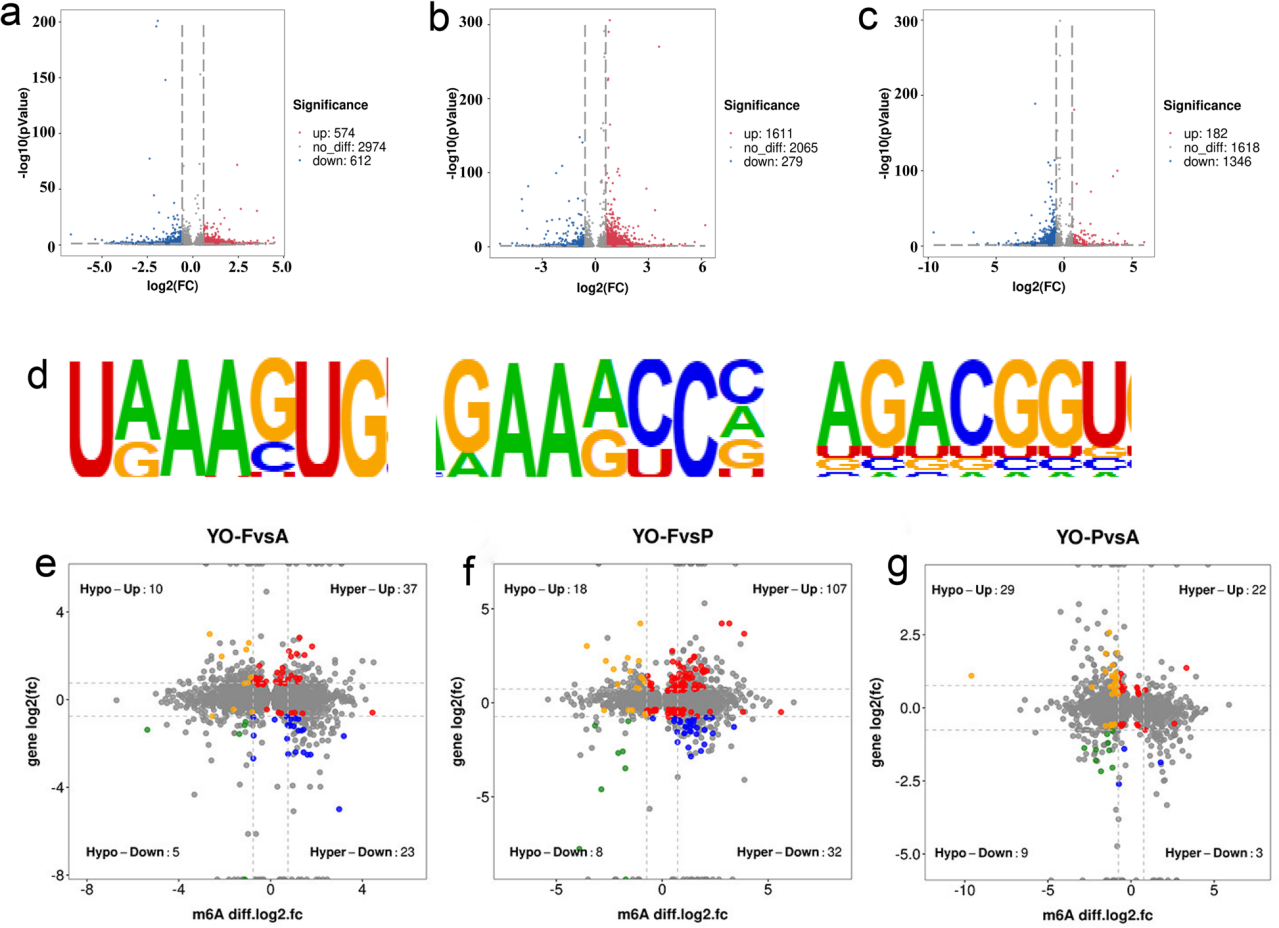
Overview of the differential methylation peaks in the YO-FvsA, YO-FvsP, and YO-PvsA comparison groups. **a** The number of hypermethylation and hypomethylation in the YO-FvsA group. **b** The number of hypermethylation and hypomethylation in the YO-FvsP group. **c** The number of hypermethylation and hypomethylation in the YO-PvsA group. **d** The motif sequences “RRACH” were identified in the YO-FvsA, YO-FvsP, and YO-PvsA groups. **e** The four-quadrant diagram of the differentially expressed genes and m^6^A methylation genes in the YO-FvsA group. **f** The four-quadrant diagram of the differentially expressed genes and m^6^A methylation genes in the YO-FvsP group. **g** The four-quadrant diagram of differentially expressed genes and m^6^A methylation genes in the YO-PvsA group

The ovarian samples obtained from the three yaks- YO-A, YO-F, and YO-P were divided into three comparison groups, YO-FvsA, YO-FvsP, and YO-PvsA. The comparative analysis of these three groups separately screened 1186, 1890, and 1528 differentially m^6^A peaks in the YO-FvsA, YO-FvsP, and YO-PvsA (Supplementary table S3). Compared to YO-A group, 574 m^6^A peaks were hypermethylated and 612 m^6^A peaks were hypomethylated in the YO-F group (Fig. 3a); compared to the YO-P group, 1611 m^6^A peaks were hypermethylated and 279 m^6^A peaks were hypomethylated in the YO-F group (Fig. 3b); compared to the YO-A group, 182 m^6^A peaks were hypermethylated, and 1346 hypomethylated m^6^A peaks in YO-P group(Fig. 3c). Overall, the differences of m^6^A in the ovarian mRNAs were the greatest between the follicular phase and pregnancy, and the smallest between the follicular phase and anestrus. The motif with high reliability in the peak region was analyzed using the motif analysis software, Homer, and the width, E-value, position frequency matrix (PFM), position-specific scoring matrix (PSSM) of each motif, and its total position information in each peak sequence were obtained. The sequence modified by m^6^A was identified as “ RRACH” (R is A/G, A is m^6^A, H is A/C/U) [17, 18]. To determine whether the identified m^6^A peaks were enriched on the common sequence of RRACH, we performed the motif prediction for each group and the differential results, and found consistent results in the differential methylation peaks of YO-FvsA, YO-FvsP, and YO-PvsA (Fig. 3d).

### The association analysis between the differential m^*6*^A peaks and differentially expressed genes

**Fig. 4:**
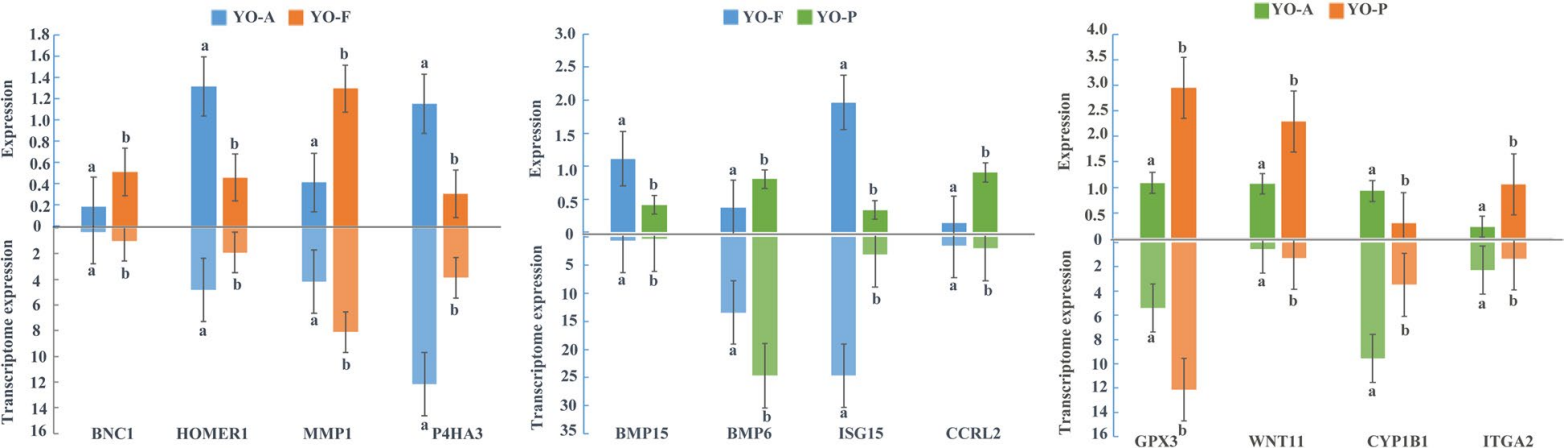
The gene qPCR results and transcriptome expression levels of *BNC1, HOMER1, MMP16, P4HA3, BMP15, BMP6, ISG15, CCRL2, GPX3, WNT11, CYP1B1,* and *ITGA2* in the YO-A, YO-F and YO-P groups

To further correlate the m^6^A modification with gene expression, we combined the MERIP-seq and RNA-seq analyses to investigate whether the degree of m^6^A methylation was related to the mRNAs of differentially expressed genes (DEGs) (Supplementary table S4). To intuitively represent the association between gene expression and m^6^A methylation, we constructed a four-quadrant diagram of the differentially expressed genes and m^6^A methylation genes. The results showed that in the YO-FvsA group (Fig. 3e), 75 genes were identified as significantly co-differentially expressed genes, including 60 hypermethylated DMRs (37 mRNAs were upregulated, 23 mRNAs were downregulated) and 15 hypomethylated DMRs (10 mRNAs were upregulated, 5 mRNAs were downregulated); A total of 165 co-differentially expressed genes were identified in the YO-FvsP group (Fig. 3f), including 139 hypermethylated DMRs (107 mRNAs were upregulated, 32 mRNAs were downregulated), and 26 hypomethylated DMRs (18 mRNAs were upregulated and 8 mRNAs were downregulated); A total of 63 co-differentially expressed genes were identified in the YO-PvsA group (Fig. 3g), including 25 hypermethylated DMRs (22 mRNAs were upregulated, 3 mRNAs were downregulated) and 38 hypomethylated DMRs (29 mRNAs were upregulated, 9 mRNAs were downregulated). We then found that the mRNAs related to ovarian and follicular development such as *BNC1, HOMER1, P4HA3, BMP15, and BMP6* in the YO-FvsA and YO-FvsP groups all showed significantly increased m^6^A levels compared to the comparison group. The transcriptome analysis showed that the *BNC1* transcription level was significantly upregulated in the estrus ovaries compared to the anestrus ovaries and pregnant ovaries, and the *BMP15* transcription level was also significantly upregulated in estrus ovaries compared to the pregnant ovaries. On the contrary, the transcription levels of *HOMER1* and *P4HA3* were significantly downregulated compared to the anestrus group, and the transcription of *BMP6* was significantly downregulated compared to the pregnant group. In the YO-PvsA group, we found the mRNA *GPX3* and *WNT11* with significantly lower m^6^A levels compared to the control group, while their transcription levels were significantly upregulated compared to the anestrus ovaries. Then, four key genes were selected from the three comparison groups, and the comparative analysis of gene qPCR results and transcriptome expression was carried out (Fig. 4). It was found that the expression trend of gene at qPCR level and transcription level was consistent, which also further verified the results of transcriptome.

### The GO and KEGG analysis of differentially methylated RNAs

To estimate the potential biological significance of m^6^A methylation in the ovary, we divided the differential methylation peaks in YO-FvsA, YO-FvsP, and YO-PvsA into hypermethylated and hypomethylated DMRs (differentially methylated RNAs). Gene Ontology (GO) and Kyoto Encyclopedia of Genes and Genomes (KEGG) were used to analyze the gene function enrichment of these DMRs with significantly changes in the methylation level of each comparison group. The GO analysis of the hypermethylated and hypomethylated DMRs in YO-FvsA group (the number of genes involved in GO item > 2, *P* < 0.05) showed that in terms of molecular function, they were significantly correlated with protein binding, ion binding, catalytic activity, and transferase activity; In terms of biological processes, the hypermethylated and hypomethylated DMRs were significantly involved in cellular processes, metabolic processes, the regulation of biosynthesis and metabolic processes; In terms of cell components, it is mainly related to organelle organization (Supplementary Fig. 1). The GO analysis of the YO-FvsP and YO-PvsA groups is similar to the results of the YO-FvsA group, as shown in the ( Supplementary Fig. 2 and 3).

The KEGG analysis of the YO-FvsA group (Supplementary Fig. 1) (the number of genes involved in the KEGG item > 2, *P* < 0.05) showed that the hypermethylated DMRs significantly participated in the selenocompound metabolism, ubiquinone and other terpenoid-quinone biosynthesis pathway, and the hypomethylated DMRs significantly participated in the Notch signal pathway, Parathyroid hormone synthesis, secretion and action, and NF-kappa B signaling pathway. The KEGG analysis results of the YO-FvsP group (Supplementary Fig. 2) showed that hypermethylated DMRs peaks were significantly enriched in adipocyte factor signaling pathway, Th17 cell differentiation, notch signaling pathway, PPAR signaling pathway, AMPK signaling pathway, and hypomethylated DMRs were significantly enriched in pyruvate metabolism, dopaminergic synapse, sphingolipid signaling pathway. The KEGG analysis results of the YO-PvsA group (Supplementary Fig. 3) showed that the hypermethylated DMRs were mainly related to AMPK signaling pathway and fatty acid metabolism, and the hypomethylated DMRs were mainly related to base excision repair and notch signaling pathway. These significantly enriched pathways may be related to special regulatory mechanisms such as hormone secretion on the ovary during estrus and follicular development. These results indicate that m^6^A is involved in multiple physiological activities of the ovary during estrus and pregnancy in yaks.

### The gene set enrichment analysis of all differentially expressed gene

**Fig. 5:**
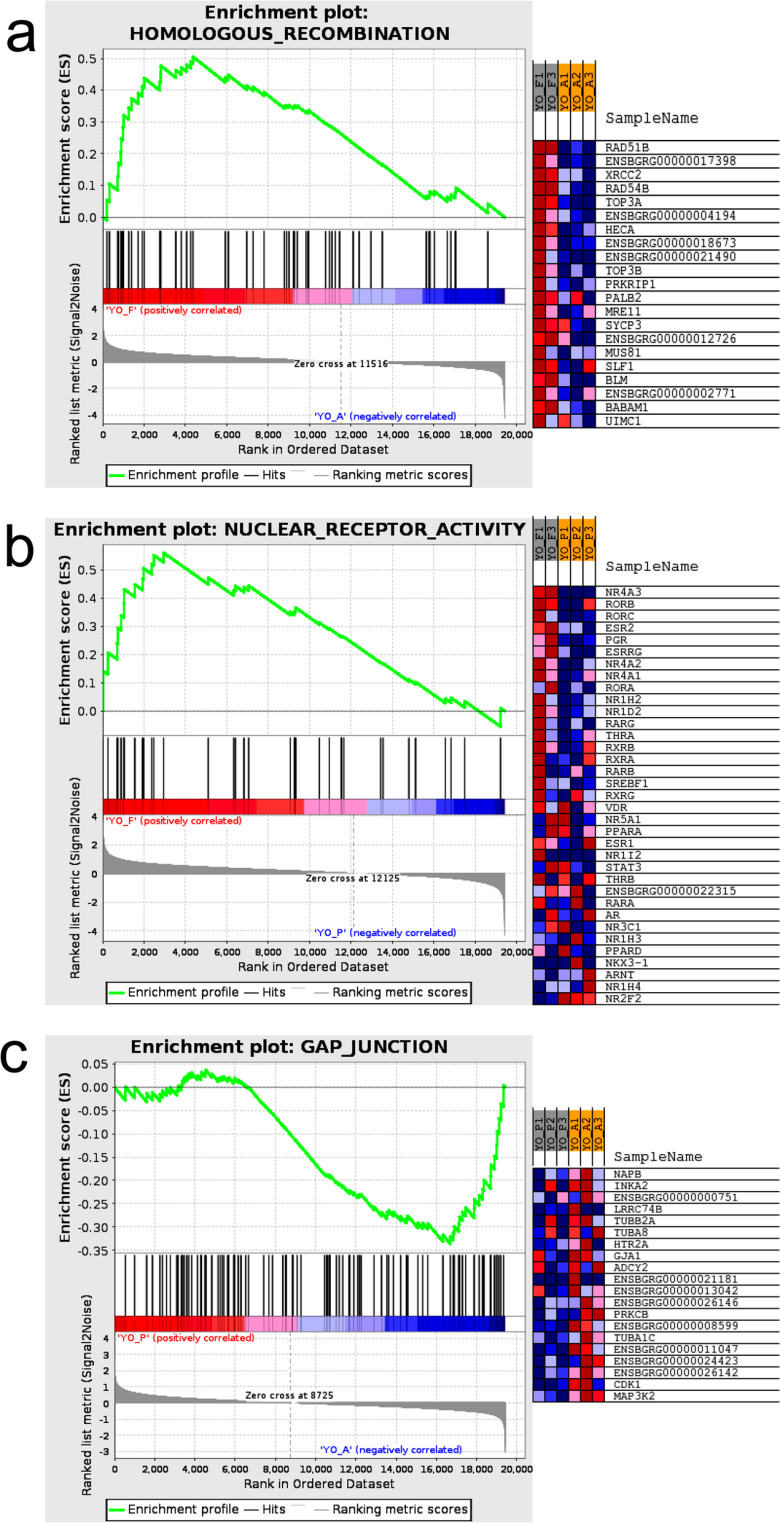
The enrichment plot of the GSEA results and the heat map of gene expression in each sample. **a** On the left is the enrichment plot of homologous recombination, on the right is the heat map of gene expression in YO-F and YO-A. **b** On the left is the enrichment plot of nuclear receptor activity, on the right is the heat map of gene expression in YO-F and YO-P. **c** On the left is the enrichment plot of gap junction, on the right is the heat map of gene expression in YO-P and YO-A

In the GSEA results, ES (enrichment score) represents the GSEA gene enrichment score, NES (normalized enrichment score) represents the normalized gene enrichment score, and FDR (false discovery rates) is the *P* value corrected for multiple hypothesis testing. Results were filtered according to *P* < 0.05 and FDR < 0.25. In the GSEA results of YO-FvsA, a KEGG gene set is related to homologous recombination, NES = 1.65; *P* = 0.002; FDR = 0.17. The gene set is upregulated in YO-F, as shown in Fig. 5a. Among the genes that significantly affect the enrichment fraction, we found SYCP3, XRCC2 and PALB2, which affect the meiosis process of oocytes. In the GSEA results of YO-FvsP, a GO gene set of nuclear receptor activity related to follicular development, it is upregulated in YO-F. Figure 5b shows the enrichment plot of nuclear receptor activity, NES = 1.68; *P* = 0.003; FDR = 0.23, the genes with higher enrichment scores include ESR2 and PGR. In the GSEA results of YO-PvsA, a KEGG gene set is gap junction (Fig. 5c), which related to mitosis, NES = -1.34; *P* = 0.05; FDR = 0.25. The gene set is upregulated in YO-A, CDK1 and GJA1 are two of its core factors.

## Discussion

The m^6^A modification, evident in various tissues reportedly regulates the gene expression and executes corresponding biological functions by regulating the RNA metabolism, splicing, degradation and translation [19]. In recent years, reversible RNA methylation modifications have led to the third wave of epigenetic modifications based on DNA and protein modifications. Many studies related to m^6^A have been conducted in the model organisms such as mice, *Arabidopsis thaliana*, and common organisms such as pigs and poultry, but there are no reports on the map of m^6^A in yak. To our knowledge, this is the first comprehensive and high-throughput study on RNA methylation in the yak ovary. Our data indicate that there is a significantly large amount of m^6^A methylation in the yak ovary during the estrus and pregnancy stages. Through further analysis, we found that m^6^A modification may play an important role in ovarian activity.

Many studies have shown that the m^6^A peaks are mainly concentrated in long exons, stop codons and 3 ′ UTR [13, 20]. Our results also showed that these peaks are mainly concentrated in the exon region. These peaks aligned to the intron and intergenic regions may be due to pre-mRNA, splicing events, incomplete genome annotation, and background noise. The methylation and demethylation of RNA start from multiple binding proteins bind to the motif at the site where the methylation occurs. The motif is essentially a biologically significant nucleic acid sequence pattern that these RNA methylation-related enzymes can recognize and bind to influence the gene expression [21]. The study of gene expression regulation mechanisms is the focus of biological research. Identifying these motifs is of great significance for the study of mechanisms regulating gene expression. According to the published data, the consistent motif “RRACH’’ was overexpressed in the m^6^A motif region [11, 22], our results also confirm it.

As the basic functional unit of the ovary, the follicle is responsible for the growth and ovulation of high-quality oocytes and produces steroid and peptide hormones which are important for reproductive physiology [23]. An enormous amount of primordial follicles are stored in the ovary of mammals from birth. In our results, the number of growing follicles in the ovaries of yaks was less in the anestrus period. With the arrival of the estrus season, the dominant follicles began to appear in the ovaries of yaks and secreted a lot of estrogens, hence, the yaks showed estrus symptoms and ovulated. There will still be a wave of non-ovulatory follicles in the ovary during pregnancy [24], but the number of such follicles is less than that in the non-pregnant state. The types of follicles present in the ovaries during pregnancy are similar to those in the anestrus period, mainly replacement follicles, and fewer selected follicles and dominant follicles. The corpus luteum is usually formed in the ovaries during pregnancy and used to synthesize estrogen and progesterone [25]. However, as the fetus develops, the corpus luteum is gradually replaced by the placental tissue, and the corpus luteum begins to shrink.

The formation, growth and function of follicles are controlled by the ovarian endocrine signal network. In this process, the hormones and cytokines strictly regulate the secretion of the gonadal hormones and expression of steroid-producing proteins in the corpus luteum and granulosa cells through various signaling pathways such as the PI3K/AKT, mTOR, transforming growth factor β, and Notch signaling pathways [26–28]. Interestingly, we also found consistent results after performing KEGG on the hypermethylated genes and hypomethylated genes of each comparison group, the Notch signaling pathway appeared in all three comparison groups. The Notch signaling plays a significant role in ovarian function, such as follicle assembly and growth, oocyte meiotic maturation, ovarian angiogenesis and steroid production [27, 28]. The expression of the Notch signal components in the mammalian ovary is conservative, which is mainly accomplished by the interaction of one of the four Notch receptors (Notch1–4) with one of the five notch ligands Jagged1 (Jag1), Jagged2 (Jag2), Delta like 1(Dll1), Dll3 and Dll4 [29]. The Notch signal has both inhibitory and stimulating effects on steroid synthesis. Wang et al. [30] reported that Notch signaling plays an inhibitory role in progesterone biosynthesis and steroidogenic protein expression in GCs. Jag1 is the most abundant Notch ligand expressed in the mouse ovary. According to Prasya and Mayo [31], the absence of Jag1 in mouse GCs results in the inhibition of granulosa cell differentiation, which shows that the expression of enzymes and factors involved in steroid biosynthesis and steroid secretion decreases, indicating that Notch signal has a stimulating effect on steroid synthesis. Conditional knockout of Notch2 in the granulosa cells and Jag1 in the oocytes leading to the formation of multiple oocyte follicles and subsequently declines the fertility of the mice [32, 33].

The results of GSEA based on dataset suggest that the gene sets are related to follicular development and oocyte meiosis. Homologous recombination, a recombination between DNA molecules that occurs during meiosis in eukaryotic oocytes, is upregulated in estrus samples, it can be seen that oocytes in estrous ovaries are undergoing meiosis and follicles are also growing and developing. Among the core expression genes in homologous recombination, studies have shown that SYCP3, XRCC2 and PALB2 can affect the process of meiosis, and then affect the growth and differentiation of oocytes [34–36]. Nuclear receptor activity is also upregulated in ovaries during estrus, yak ovaries secrete steroid hormones such as estradiol and aldosterone. After entering cells, steroid hormones will activate nuclear receptors, thereby changing the expression of hundreds of specific target genes in the genome [37, 38]. In the nuclear receptor activity gene set, there are two highly expressed nuclear receptor genes Estrogen receptor 2 (ESR2) and Progesterone receptor (PGR). ESR2 plays a key role in folliculogenesis and ovulation, and can regulate granulosa cell genes necessary for follicular maturation and ovulation [39]. PGR is highly expressed in granulosa cells of preovulatory follicles and plays an important role in successful ovulation in the ovary [40]. Gap junction is significantly upregulated in anestrus ovaries, cyclic GMP (cGMP) produced in granulosa cells diffuses into oocytes through gap junctions, thereby maintaining meiotic prophase arrest [41]. Studies have shown that oocytes maintain meiotic arrest mainly through inhibitory phosphorylation of CDK1 (cyclin-dependent kinase 1) [42]. GJA1 (also known as connexin 4) is the most abundant gap junction protein in the mammalian ovary, and GJA1 has been studied as a potential gene marker associated with oocyte maturation [43]. The development of mammalian follicles is coordinated by a complex interaction of multiple signals, including gonadotropins, paracrine/autocrine, and paracrine signals [44]. Through the analysis of transcriptome and differential peaks, some genes related to ovarian function were found, such as *BNC1, HOMER1, BMP15, BMP6, GPX3*, and *WNT11*. BNC1 appears to be a member of the mammalian maternal effect gene family. Previous studies have detected the BNC1 protein in adult ovarian tissue, especially in the oocytes that are in the secondary follicular phase and the ovulating oocytes with nuclear-cytoplasmic expression patterns [45]. The female mouse model with *BNC1* frame-shift mutation showed infertility, due to the significantly increased serum follicle-stimulating hormone, and reduced ovary with a declined number of follicles [46]. Our results showed that the *BNC1* m^6^A levels were significantly increased in the ovaries during estrus and the expression of the mRNAs was also significantly higher than those in the ovaries during anestrus and pregnancy. Therefore, we speculated that the mRNA expression of the m^6^A modified *BNC1* gene is upregulated during estrus and ovulation, which promotes oocyte development and follicle formation. A study by Zhang et al. speculated that BNC1 might affect oocyte genesis through the oocyte-derived growth factor/signal transduction and PI3K pathway [45].The bone morphogenetic proteins 6 (BMP6) and 15 (BMP15) are transforming growth factors-β (TGF-β) superfamily, secreted by the oocytes [47]. BMP15 has been shown to play an important role in mammalian ovarian and follicular development, including promoting the proliferation of the granulosa cells (GCSs) and steroid production, controlling the ability of oocytes, and ovulation [48, 49]. BMP6 is expressed in the oocytes and granulosa cells of healthy follicles and inhibits the activity of FSH by inhibiting the activity of adenylate cyclase and lead to the “selection” of dominant follicles [50]. In addition, BMP6 also prevents bovine cumulus cell apoptosis and preserves the normal oocyte quality. In our study, the m^6^A and mRNA levels of *BMP15* in the estrus ovaries were significantly higher than those in pregnancy. Therefore, the m^6^A methylation of BMP15 may play a potential role in promoting the growth and maturation of yak follicles.

We found that the expression levels of *METTL3* and *FTO* in the anestrus ovary were significantly higher than those in the estrus and pregnancy ovaries, instead, the expression of readings *YTHDF1, YTHDF2*, and *YTHDC2* during the estrus and pregnancy were significantly higher than those in the anestrus. Studies have shown that METTL3, FTO, YTHDF2, and YTHDC2 are related to regulating mRNA degradation, mRNA stability, and gene expression [8, 51]. In estrous ovaries, the level of m^6^A and transcripts of BNC1 and BMP15 were up-regulated, indicating that methylase promoted the expression of mRNAs. The m^6^A levels of HOMER1, P4HA3, and BMP6 in the ovary during estrus were significantly up-regulated compared with the anestrus and pregnant ovary. However, in transcription level, the HOMER1 and P4HA3 were significantly down-regulated compared with the anestrus ovary, and the transcription level of BMP6 was significantly lower than that of the pregnant ovary. This phenomenon maight be related to the regulation of the degradation of mRNAs and the inhibition of gene expression levels by methylases. However, the specific methylase and the regulatory mechanism are still unclear.

## Conclusions

In summary, this study analyzed the m^6^A methylation modification in the ovary tissues of the yak during the anestrus, estrus, and pregnancy. The differences in the expression of *METTL3*, *METTL14*, *FTO*, and other enzymes and the functional enrichment analysis of the methylated genes suggest that m^6^A methylation might influnce the regulation of the yak follicles development and the expression of sex hormone secretion-related genes. This study shows that *BNC1, HOMER1, BMP15, BMP6, GPX3,* and *WNT11* are most likely to play a key role in regulating the growth and development of follicles. In addition, the data obtained by the high-throughput sequencing provides a foundation for future research on the role of m^6^A methylation in the process of animal estrus and ovulation.

## Methods

### Sample collection

The ovarian tissues were collected from three healthy yaks of the same age during three different periods of the year. The samples were separately collected during the anestrus in April (A1, A2, A3), the follicular (also as estrus) phase in August (F1, F2, F3), and the pregnancy period in December (P1, P2, P3). Identification of the yak estrus was performed as described by Zi et al. [52]. The ovarian tissue was divided into two parts, one part was fixed in 10% neutral formaldehyde for making tissue sections, the other part was immediately stored in liquid nitrogen for RNA isolation. All the samples were collected from the Menyuan Hui Autonomous County, Haibei Tibetan Autonomous Prefecture, Qinghai Province, China (37°39〞N, 101°62〞E). All ovarian samples were collected from euthanized yaks within 1 h.

### HE staining

The fixed-tissues were dehydrated, embedded, and sliced using a fully automatic dehydrator, the sections were dewaxed and stained by conventional HE staining, sealed with neutral gum. The above specimens were carried out according to the pathological examination standard operation procedure (SOP). The microscopic examination of the slice was captured using the Pannoramic 250 digital slice scanner (DRNJIER, Ji Nan, CHINA).

### RNA isolation and purification

The total RNA was isolated and purified using the TRIzol reagent (Invitrogen, Carlsbad, CA, USA) according to the manufacturer's instructions. The amount and purity of the RNA samples were quantified using the NanoDrop ND-1000 (NanoDrop, Wilmington, DE, USA). Then the concentration and integrity of each RNA sample was assessed by Bioanalyzer 2100 (Agilent, CA, USA) and verified by agarose electrophoresis. The RNA that meets the conditions of concentration > 50 ng/μL, Rin > 7.0, OD260 / 280 > 1.8, total RNA > 50 μg, is used for downstream experiments.

### RT-qPCR

The extracted RNA was reverse transcribed to obtain cDNA using the *Evo M-MLV* RT Kit with gDNA Clean for qPCR ‖ (AG, Hunan, China). The primer synthesis template of the target gene is shown in Supplementary table S5. The real-time quantitative real-time PCR (RT-qPCR) was performed using the SYBR Green master mix (Yeasen, Shanghai, China) and the LightCycler System (CFX96TM Optics Module, Singapore). RT-qPCR experiment was performed in triplicate on three samples in the periods of anestrus and pregnancy, and on two samples in the period of estrus. The reaction system and PCR procedure refer to the methods of Wang et al. [53]. p The data are expressed as mean ± SE (*n* = 3). The *Glyceraldehyde-3-phosphate dehydrogenase (GAPDH)* gene was used as an internal reference gene, and the expression of mRNA was normalized by the 2^−ΔΔCT^ method. All the statistical analyses were calculated using the ANOVA program in the SAS 9.4 statistical software.Statistically significant difference were considered as *P* < 0.05.

### The methylated RNA immunoprecipitation sequencing (MeRIP-Seq)

The Poly (A) RNA was purified from 50 μg total RNA using Dynabeads Oligo (dT) (Thermo Fisher, CA, USA) through two rounds of purification. The fragmented RNA was premixed with the Dynabeads Antibody Coupling Kit (Thermo Fisher, CA, USA) and m^6^A antibody (Synaptic Systems, Germany) in IP buffer and incubated for 2 h at 4 °C. The IP RNA was reverse-transcribed to synthesize the cDNA using the SuperScript™ II Reverse Transcriptase (Invitrogen, USA). Then the two-strand synthesis was performed with RNase H (NEB, USA) using the *E. coli* DNA polymerase I (NEB, USA) to synthesize U-labeled second-stranded DNAs. At the same time, the dUTP Solution (Thermo Fisher, CA, USA) was incorporated into the two strandsand the size of the fragment was screened and purified using the AMPureXP beads. The U-labeled second-stranded DNAs were digested using a heat-labile UDG enzyme (NEB, USA), then amplified by PCR to form a cDNA library of 300 ± 50 BP. At last, the 2 × 150 bp paired-end sequencing (PE150) was performed on an Illumina Novaseq™ 6000 (LC-Bio Technology CO., Ltd., Hangzhou, China) following the vendor's recommended protocol.

### Data analysis

The Fastp software was used to remove the reads of the IP and input samples that contained splices, repetitive sequences and low-quality sequences [54] with default parameters. The map reads to the reference genome of *Bos grunniens* (Version: v101) [55] by HISAT2. The R package exomepeak [56] was used for peak calling analysis and gene difference peak analysis for the bam files obtained from the IP samples and input samples. According to all the unique comparison reads in this region, the Poisson distribution model was used to test and calculate the *p*-value of the candidate peak region. The default *p*-value < 0.05, the area smaller than the *p*-value is considered to be a peak. The called peaks were annotated by intersection with the gene architecture using R package ChIPseeker [57], MEME [58] and HOMER were used for motif analysis. The StringTie software [59] was used to perform gene assembly and quantification by using the quantification method FPKM (total exon fragments /mapped reads (millions) × exon length (kB)). The differentially expressed mRNAs were selected with log2 (fold change) > 0.58496 or log2 (fold change) < -0.58496 and *p* value < 0.05 using the R package edgeR [60]. We conduct go analysis through G: profiler website [61], and KEGG analysis through Kobas 3.0 website [62, 63]. The gene expression matrix of each group of samples was obtained according to the sequencing results, and GSEA (Gene Set Enrichment Analysis) was performed on the expressed genes using GESA v1.8. The gene set used custom GO and KEGG gene sets, process the data by de-extremum and Z-score.

## Supplementary Information

**Additional file 1:** **Supplementary Figure 1.** The GO and KEGG analysis of the hypermethylated and hypomethylated DMRs in YO-FvsA group.

**Additional file 2:** **Supplementary Figure 2.** The GO and KEGG analysis of the hypermethylated and hypomethylated DMRs in YO-FvsP group.

**Additional file 3:** **Supplementary Figure 3.** The GO and KEGG analysis of the hypermethylated and hypomethylated DMRs in YO-PvsA group.

**Additional file 4:** **Table S1.** The statistics results of sequencing data and reference genome reads in YO-A, YO-F, and YO-P samples.

**Additional file 5:** **Table S2.** The annotation of peaks on the genome including the detailed location  and length information in YO-A, YO-F, and YO-P samples.

**Additional file 6:** **Table S3.** The differential m6A peaks and annotations were screened in YO-FvsA, YO-FvsP, and YO-PvsA groups.

**Additional file 7:** **Table S4.** The combined analysis results of MERIP-seq and RNA-seq in YO-FvsA, YO-FvsP, and YO-PvsA groups.

**Additional file 8:** **Table S5.** Primer sequence of target gene in qPCR experiment.

## Data Availability

The dataset supporting the conclusions of this article is available in the GEO repository, unique persistent identifier and hyperlink to datasets in https://www.ncbi.nlm.nih.gov/geo/query/acc.cgi?acc=GSE180401. The data supporting the conclusions of this study are available within the Additional files.

## Abbreviations

m^6^A: N6-methyladenosine
CDS: Coding sequence
3′UTR: 3′Untranslated region
MeRIP-seq: Methylated RNA immunoprecipitation sequencing
DMRs: Differentially methylated RNAs
GO: Gene Ontology
KEGG: Kyoto Encyclopedia of Genes and Genomes
DEGs: Differentially expressed genes
