# Supplementary figures and images for: The transcriptome-wide N6-methyladenosine (m6A) map profiling reveals the regulatory role of m6A in the yak ovary
Source: BMC Genomics. 2022 May 11;23:358. doi: 10.1186/s12864-022-08585-7 (PMC9092806; doi:10.1186/s12864-022-08585-7)

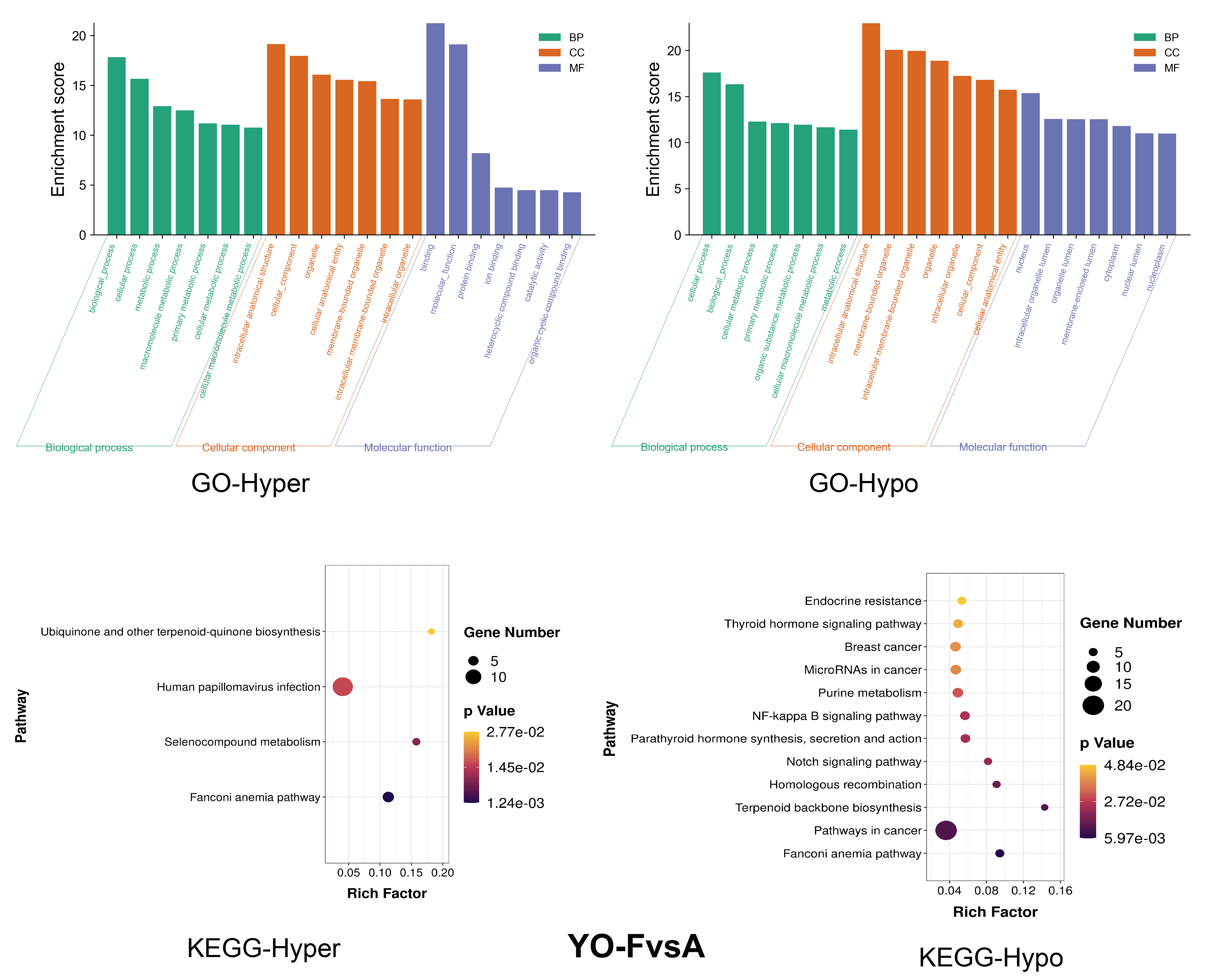

Supplement: Supplementary file 1 — Additional file 1: Supplementary Figure 1. The GO and KEGG analysis of the hypermethylated and hypomethylated DMRs in YO-FvsA group. [file 12864_2022_8585_MOESM1_ESM.tif]

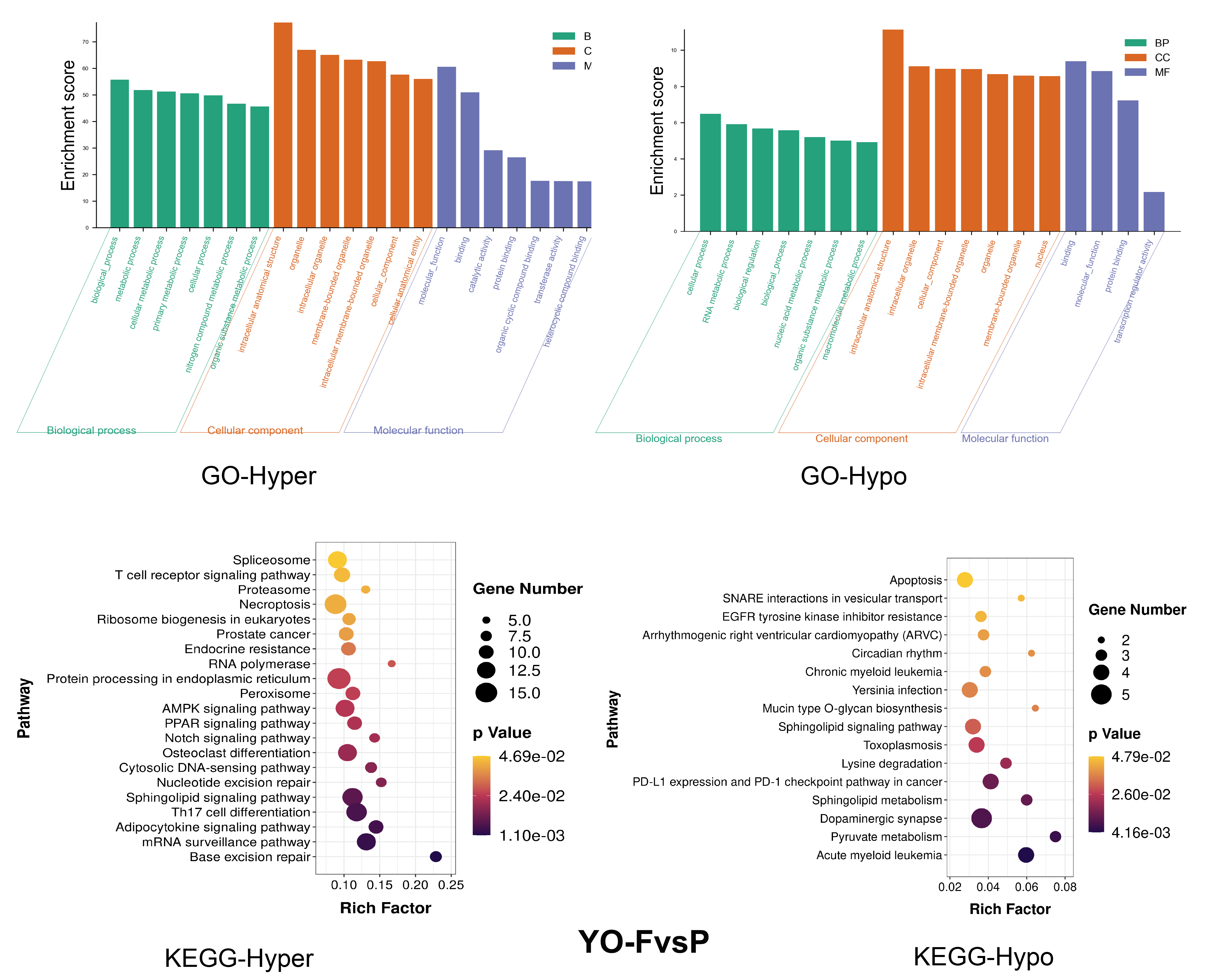

Supplement: Supplementary file 2 — Additional file 2: Supplementary Figure 2. The GO and KEGG analysis of the hypermethylated and hypomethylated DMRs in YO-FvsP group. [file 12864_2022_8585_MOESM2_ESM.tif]

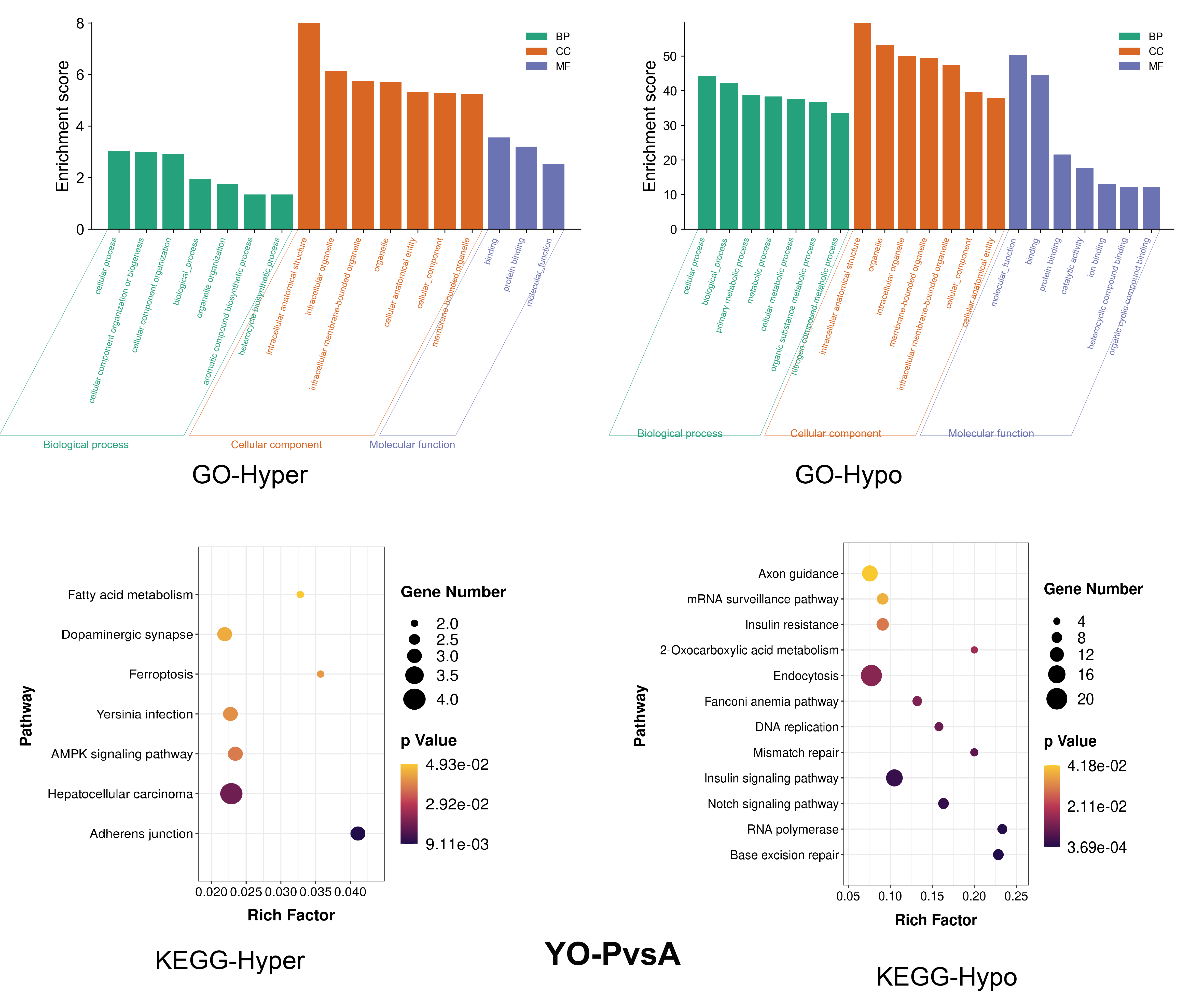

Supplement: Supplementary file 3 — Additional file 3: Supplementary Figure 3. The GO and KEGG analysis of the hypermethylated and hypomethylated DMRs in YO-PvsA group. [file 12864_2022_8585_MOESM3_ESM.tif]
